# One Health implementation: A systematic scoping review using the Quadripartite One Health Joint Plan of Action

**DOI:** 10.1016/j.onehlt.2025.101008

**Published:** 2025-03-02

**Authors:** Adriana Milazzo, Jingwen Liu, Priyanka Multani, Sandra Steele, Elizabeth Hoon, Anne-Lise Chaber

**Affiliations:** a School of Public Health, The University of Adelaide, South Australia 5000, Australia; b Melbourne Veterinary School, The University of Melbourne, Victoria 3010, Australia; c School of Animal and Veterinary Sciences, The University of Adelaide, South Australia 5000, Australia

**Keywords:** One Health, Operationalization, Implementation, Quadripartite, One Health Joint Plan of Action, Theory of Change

## Abstract

**Background:**

One Health (OH) recognizes the interconnectedness of humans, animals, and their shared environment and mobilizes multiple sectors to achieve sustainable and optimal health outcomes. We synthesized current OH implementations at global, regional, national, and local community levels using the OH Joint Plan of Action (OH JPA) developed to enhance best practices in OH implementation.

**Methods:**

We applied the OH JPA Theory of Change, supported by three pathways mapped out across six action tracks to guide our review. Searches were conducted in PubMed, Embase and Scopus. Eligibility of studies was based on implementation of OH undertaken across two or more sectors within the human-animal-environment interface. Selection was guided by the PRISMA-ScR.

**Results:**

Of the 54 studies, 77 unique programs reported OH implementations, and of these, 42 (54.5 %) involved human and animal sectors only. No studies involved the environment sector and its impact on human-environment or animal-environment. The majority (90.9 %) of programs incorporated at least one aspect of policy, legislation, advocacy and financing (Pathway 1). Capacity development, community engagement, multisectoral coordination, collaboration or communication was identified in 96.1 % of programs (Pathway 2). Data, evidence and knowledge featured in 60 % of OH initiatives (Pathway 3).

**Conclusion:**

Pathway 2 was the most engaged pathway building the foundation for OH implementation and action. There is opportunity for further growth concerning community engagement, monitoring and evaluation strategies with enhanced future investment for implementation of community-centric and risk-based solutions. Furthermore, it is important to foster better understanding of environmental issues and to build capacity for the environment sector to be better represented in the implementation of OH.

## Highlights

• OH implementations heavily focused on collaboration between human and animal sectors.
• Greater representation of pathway 1 and pathway 2 implementation.
• Low-middle income countries supported by wider international programs.
• Lack of involvement from local communities and Indigenous groups on OH implementation.

## Graphical abstract

**Figure:**
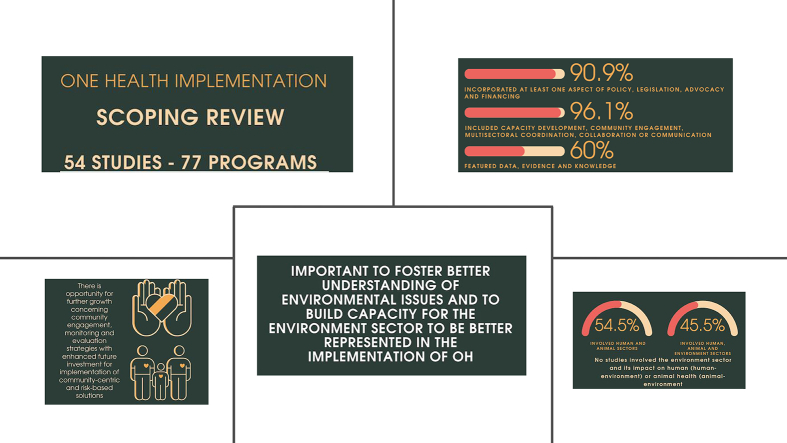

## 1 Introduction

The One Health (OH) concept was recognized long before the term was first coined in 2004 by multidisciplinary experts at a symposium convened by the Wildlife Conservation Society [1]. The symposium, largely driven by the 2002–2004 Severe Acute Respiratory Syndrome (SARS) pandemic and subsequent Highly Pathogenic Avian Influenza (HPAI) H5N1, highlighted the movement of emerging infectious diseases (EIDs) among humans, domestic animals, and wildlife [2]. Following this, an unprecedented Tripartite agreement released in 2008 by the World Health Organization (WHO), World Organization for Animal Health (WOAH, formerly OIE), and the Food and Agriculture Organization of the United Nations (FAO) aimed to reduce the risk of avian influenza and other EIDs through shared responsibility in areas where humans, animals, and ecosystems intersect [3].

More recently, the Tripartite has evolved into a Quadripartite partnership with the formal inclusion of the United Nations Environment Programme (UNEP) [4]. This reaffirms the importance of environmental factors in OH collaboration, particularly against a worsening global climate crisis and the vulnerabilities in global health security exposed by the COVID-19 pandemic [5,6].

One Health aims to sustainably balance and optimize the health of humans, animals and ecosystems. The shared need for safe food, water, energy, and air, emphasizes a holistic and systems-based approach. Bringing together various sectors, disciplines, and communities across different levels of society is required to address infectious diseases, and more broadly non-infectious diseases driven by human activities and environmental degradation. Increasing population densities, accelerating climate change, and expanding interconnectedness at the human-animal-environment interface intensify these health threats [7]. Human-induced diseases and the role of human behavior in disease transmission and environmental harm, requires a comprehensive preventative approach that includes the health of the environment. Preventing zoonoses is substantially more cost-effective than managing diseases and mitigating their economic impacts, reinforcing the need for proactive measures [8,9].

There is a growing awareness of the need to operationalize OH underpinned by political commitment and financial support, to collectively combat health risks and achieve the optimal health of humans, animals, and ecosystems [10]. Consequently, there has been a rapid growth in initiatives advocating for OH, alongside the expansion of OH networks over recent decades [6]. These efforts have strengthened collaboration across sectors at global, regional, national, and local levels. Nevertheless, a critical gap remains in evidence of how OH has been implemented. Such information is crucial for identifying opportunities and challenges in OH practices and in evaluating the impact of OH interventions and their sustainability across regions [11]. Determining which aspects require investment to ensure more effective execution is warranted. Sharing the learnings around implementation of OH activities adds evidence to where investments should be prioritized.

Heightened awareness about OH and formation of the Quadripartite has led to the recent collaborative development of the One Health Joint Plan of Action (OH JPA) Theory of Change (ToC) [10]. The OH JPA is a framework for action addressing health across the three domains (human, animal, environment) and is structured on the ToC. The ToC guides the implementation of the OH JPA and provides an analytical approach to how and why an intervention will lead to the intended outcome and impacts. Exploring the values, beliefs and role of stakeholders and systems provides an understanding of where, and how organizations can collaborate to achieve change and impact [12]. The OH JPA ToC framework is structured across three pathways and six action tracks to achieve “sustainable health and food systems, reduced global health threats and improved ecosystem management” [10]. This can be achieved through a OH approach by strengthening collaboration, communication, capacity building and coordination within and across the three pathways. Pathway 1 concerns policy, legislation, advocacy and financing, pathway 2 has a focus on organizational development, implementation and sectoral integration and pathway 3 supports data, evidence and knowledge. The six action tracks detail specific objectives, activities, deliverables and timelines to drive future changes in the prevention, prediction, and response to health threats, while also contributing to sustainable development. Despite these advancements, there remains a limited understanding of how OH has been implemented at global, regional, national, and local levels. This review aims to address this gap by identifying and analysing the implementation of OH activities reported in the literature using the OH JPA ToC.

## 2 Methods

The study protocol was registered with the Open Science Framework (https://doi.org/10.17605/OSF.IO/NJUXZ) and follows the Preferred Items for Systematic Reviews and Meta-Analysis guidelines extension for Scoping Reviews (PRISMA-ScR) [13]. A systematic scoping review was conducted to synthesize evidence on the actions implemented for operationalizing a OH approach. In this study, we define the operationalization of OH as translating the concept of integrating human, animal, and environmental health into practical strategies, policies, and actions. It encompasses the implementation of collaborative frameworks, interdisciplinary partnerships, and measurable interventions aimed at achieving improved health outcomes across these interconnected domains. We used the OH JPA, a framework designed to address multidimensional global health threats, as a tool to assess the operationalization of OH activities.

### 2.1 Search strategy and selection criteria

We searched PubMed, Embase, and Scopus databases for studies reported on OH implementation using the key terms: “Operation” OR “Implement” OR “Action”, paired with “One Health” OR “human health” OR “animal health” OR “environment”. The complete search strategy is provided in Table A1 (Appendix A, p.1). Publications in English from database inception until June 20th, 2023 were identified. Grey literature was restricted to reports from the OH Quadripartite collaboration. We also identified new relevant studies via forward snowballing used to find other publications cited in the reference list of the original article. Studies were considered eligible if they clearly indicated specific OH actions undertaken across more than two sectors within the human-animal-environment interface and within at least one of the three OH JPA pathways. We excluded studies that only advocated for OH concepts, such as proposals or guidelines, without demonstrating practical application in real-world OH initiatives. This consideration ensured that our review focused on mapping and evaluating tangible OH implementations rather than solely on theoretical discussions.

Retrieved studies were imported into Rayyan QCRI to screen titles and abstracts for their relevance against the eligibility criteria [14]. Full-text screening was performed independently by three investigators (JL, PM, SS). Disagreements on selection of studies (16.8 %) were resolved through discussion and consultation (AM, A-LC). Studies excluded during full-text screening, along with the reasons for their exclusion, is outlined in Table A2 (Appendix A, pp. 2–3).

### 2.2 Data extraction and analysis

A data extraction template (Fig. A1, Appendix A, pp. 4–9) was collaboratively designed by our team of OH researchers, with expertise in human health, veterinary medicine, and environmental epidemiology. We extracted information from the included studies on author, year, location and study type. Authors' key insights into the specific OH initiatives and actions with clear categorization and analysis of OH implementation was mapped against the OH JPA ToC. Three investigators (JL, SS, PM) independently extracted data into Covidence (https://www.covidence.org/), with 50 % of the studies cross-checked by other investigators (AM, A-LC, JL). Studies and data sources were descriptively analyzed and presented in tables and maps, with the findings summarized using a narrative synthesis approach.

## 3 Results

**Fig. 1:**
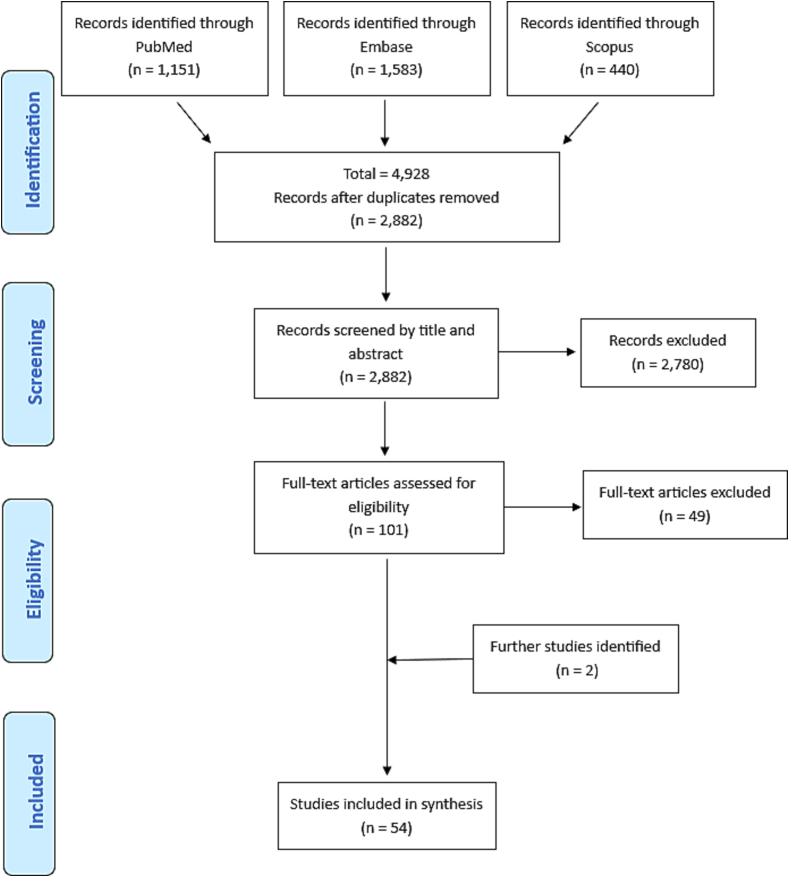
PRISMA-ScR flow chart.

In our initial systematic search across the three databases, we identified 4928 studies. After removing duplicates and title and abstract screening, we reviewed 101 studies for full-text eligibility. After excluding 49 studies that did not report on OH implementation, we identified 52 studies that met the inclusion criteria. Additionally, two studies were included through forward snowballing. In total, 54 studies were included in our analysis (Fig. 1).

### 3.1 One Health program characteristics

**Fig. 2:**
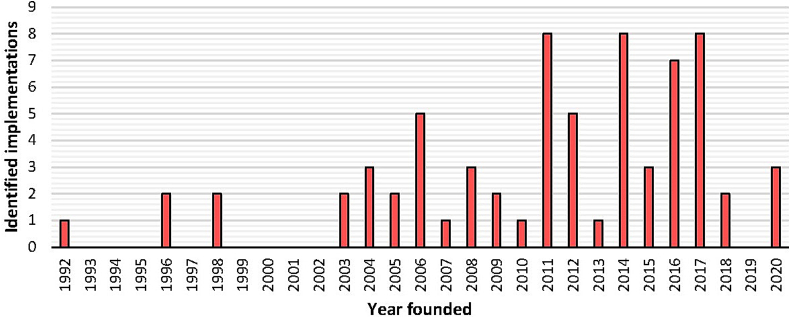
Implementation of One Health activities reported by year.

Within the 54 included studies, we identified 77 unique programs reported to have implemented OH initiatives (Table 1). Fifty six percent of these 54 studies were funded by or led by researchers from high-income countries, examples include the USA, [[15], [16], [17]] Australia, [[18], [19], [20]], UK [21,22], and European countries [[23], [24], [25]]. Of the 77 identified OH programs, 60 (77.9 %) were implemented in low-middle-income countries (LMIC) or as part of international OH initiatives. Sixty-five percent of the 77 programs commenced after 2010, with notable spikes in OH implementation reported in 2011, 2014 and 2017 (Fig. 2).

**Table 1:** Study (*n* = 54) characteristics and identified unique One Health programs (*n* = 77).

Study number (n = 54)	Author(s) by year and reference number []	OH programs by location (n = 77)	Geographic scale	OH programs highlights (n = 77)
1	Zinsstag et al., 2023 [6]	1. Côte d'Ivoire, Africa 2. UK 3. Bangladesh, Asia 4. Kenya, Africa 5. Switzerland, Europe	National	1. Côte d'Ivoire has established intersectoral collaboration between human and animal health sectors, focusing on local concerns regarding animal bite victims rather than theoretical or emerging risks of global interest. 2. The UK's OH approach, praised for its exemplary collaboration between public health and veterinary officials, is recognized as a best practice by formalized groups like the Human Animal Infections and Risk Surveillance Group (HAIRS). 3. OH Bangladesh is a collaborative organization addressing health issues at the human-animal interface with a strategic framework and action plan, supported by government and international partners, and operates a secretariat at the Institute of Epidemiology and Disease Control Research (IEDCR). 4. Kenya has established strong multisectoral and multidisciplinary teams for human and animal health at the national level, though such integration is weaker at subnational levels. There are plans and some data sharing for integrated surveillance and risk communication, comprehensive multisectoral coordination and implementation, this is limited for non-zoonotic OH issues. 5. Switzerland has robust OH capacity for zoonotic disease management, supported by a detailed roadmap, a legal subsidiary body for coordination, and strong collaboration among federal and cantonal agencies.
2	Nyokabi et al., 2023 [30]	6. Ethiopia, Africa	National	6. The OH Implementation in Ethiopia has been slow and incomplete, with animal and human health systems operating independently. Despite the Ethiopian government's increased investment in strengthening collaboration, formal and legal linkages are still absent.
3	de la Rocque et al., 2023 [29]	7. Multi-countries 8. Multi-countries 9. Eastern Mediterranean Region	Global Regional Regional	7. Beginning in 2010, the Tripartite established strategic directions to coordinate global activities and strengthen capacities to address health risks at the human-animal-environment interface, promoting a shared global vision and a coordinated approach for all countries. 8. The National Bridging Workshop (NBW) program facilitates initial planning for coordination between human and animal health sectors, conducted in over 35 countries. This enables joint review of International Health Regulation (IHR) – Monitoring and Evaluation (MEF) and Performance of Veterinary Services (PVS) Pathway results and agreement on specific, time-bound actions to improve coordination, resulting in a detailed, consensual roadmap of prioritized national activities. 9. The Tripartite has leveraged IHR-MEF and NBW findings to develop regional OH frameworks, such as the WHO's Eastern Mediterranean Region (EMRO) OH operational framework for action on zoonotic diseases, providing practical activities to optimize resources and strengthen capabilities for addressing health challenges collaboratively.
4	Belot et al., 2021 [31]	8. 35 countries	Regional	Same as 8. above
5	Mahrous et al., 2020 [26]	9. Eastern Mediterranean Region	Regional	Same as 9. above
6	Woolaston et al., 2022 [32]	10. Australia, Oceania 11. Queensland, Australia 12. Victoria, Australia	National Local Local	10. Individual governmental departments and non-government organizations lead OH policy by co-funding research, prioritizing OH in the national AMR Strategy, integrating OH into various strategies, and developing missions on Infectious Disease Resilience and AMR. 11. OH initiatives for managing zoonotic incidents. 12. OH approach to Q-fever.
7	Song et al., 2022 [33]	13. Hong Kong, Asia	Local	13. The Hong Kong Strategy and Action Plan on Antimicrobial Resistance 2017–2022 (HKSAP) aims to tackle AMR by strengthening data collection and surveillance under the OH concept and enhancing inter- and cross-sectoral collaboration.
8	Nguyen-Viet et al., 2022 [34]	14. Vietnam, Asia	National	14. Over two decades, Vietnam has applied a OH approach to address emerging infectious diseases of animal origin, focusing on managing zoonoses, food safety, and AMR through training, policy, and research initiatives.
9	Mwangi DK., 2022 [35]	15. Kenya, Africa 16. Nthongoni, Eastern Kenya, Africa	National Local	15. The OH approach in Kenya started in 2005 with a national taskforce, later evolving into the Zoonotic Technical Working Group and Zoonotic Disease Unit (ZDU). These bodies identified priority zoonoses, developed strategic plans, conducted joint surveys, and initiated prevention strategies for rabies and brucellosis. 16. The Rabies Prevention and Control program in Nthongoni trained young people to use mobile phones for reporting rabies cases in humans, animals, and wildlife.
10	Lota et al., 2022 [36]	17. Philippines, Asia	National	17. Activities include improving laboratories and surveillance, ensuring safe antimicrobial access, regulating their use, preventing infections, promoting AMR research, and raising awareness through education and communication.
11	Innes et al., 2022 [37]	18.Thailand, Asia	National	18. Thailand's federal government piloted an avian influenza surveillance system integrating human, animal, and environmental sectors to monitor influenza A viruses in humans, waterfowl, and poultry across the central level and four provinces.
12	Humboldt-Dachroeden et al., 2022 [38]	19. Italy, Europe 20. Sweden, Europe	National National	19. The OH approach involves distinct institutional and government set-ups promoting cross-sectoral collaboration, with specific agencies and task distributions established to enhance coordination. 20. Integrating the food agency with public health and veterinary agencies in Sweden improves coordination and increases OH outputs through enhanced collaboration.
13	Perez Arredondo et al., 2021 [25]	21. Ghana, Africa 22. India, Asia	National	21. OH implementation includes legal framework for the control and management of animal and zoonotic diseases, strategic plan for rabies elimination, parallel surveillance systems and response to avian influenza outbreaks. 22. In India, OH implementation has involved coordinated efforts between national and local agencies to address rabies, avian influenza, and climate-related health challenges, incorporating international frameworks and focusing on surveillance, vaccination, training, and collaborative response strategies.
14	Mariappan et al., 2021 [39]	23. Malaysia, Asia	National	23. Malaysia's OH implementation programs encompass antimicrobial stewardship, animal and food safety regulations, academic and professional collaborations on zoonotic diseases and AMR, and environmental initiatives for safe drug disposal.
15	Marchino et al., 2021 [40]	24. Northern Italy (Emilia-Romagna, Lombardy, Piedmont), Europe	Local	24. Regional health authorities have implemented integrated West Nile virus surveillance following a OH approach, based on collaboration between human, animal and environmental health institutions.
16	Joshi et al., 2021 [41]	25. 11 countries, Asia and Africa	Regional	25. A donor-funded program aims to strengthen multisectoral coordination on AMR in 11 countries to advance the objectives of the Global Health Security Agenda.
17	Achi et al., 2021 [42]	26. Nigeria, Africa	National	26. Nigeria's OH implementation focuses on increasing AMR awareness, building a OH surveillance system, enhancing infection prevention and control, promoting rational antimicrobial use, and researching alternatives, with situational analyses of antimicrobial-resistant pathogens from various sources.
18	Ogyu et al., 2020 [43]	27. Hong Kong, Asia	Local	27. Strengthen knowledge through surveillance and research and build laboratory capacity to support surveillance activities in both human and animal sectors.
19	Nambiar P., 2020 [44]	28. India, Asia	National	28. A solution-based approach targeting specific threats and outbreaks, described as a 7C process: communication, cooperation, collective action, collaboration, continual reporting, critical review, and crisis control, involving human, animal, and environmental health sectors led by the district administrator.
20	Mtui-Malamsha et al., 2020 [45]	29. Tanzania, Africa	National	29. Tanzania launched the OH Coordination Desk (OHCD) to bridge gaps between the centralized public health system and the decentralized, privatized animal health system, pilot testing OH Rapid Response Teams (OHRRTs) in selected districts and regions to facilitate relevant training.
21	Häsler et al., 2020 [46]	30. Ireland, Europe 31. New South Wales, Australia 32. Sub-Saharan Africa	National Local Regional	30. Chaired by a veterinary epidemiologist, the interdisciplinary OH team ensured national mathematical models (COVID-19 modeling support) were grounded in robust biological understanding, crucial for adapting to rapidly changing epidemiological data. 31. NSW Department of Primary Industries (DPI) and the Animal Health Committee developed science-based animal health policies for COVID-19, supporting agriculture and ensuring food supply continuity. The response emphasized the importance of a well-resourced OH approach and effective communication. 32. A university-led network across African countries, promotes OH approaches in education. During COVID-19, it supported national responses by providing a platform for professionals and students to access current information and discuss response strategies through virtual sessions, involving experts in various health fields.
22	Buregyeya et al., 2020 [47]	33. Uganda, Africa	National	33. Uganda's OH achievements include multi-sectoral disease response initiatives, strong political leadership, establishment of the OH framework and technical working group, the Zoonotic Diseases Coordination Office, prioritization of zoonotic diseases, and integration of the OH approach in academia.
23	Atusingwize et al., 2020 [48]	34. Makerere University, Uganda	Local	34. Makerere University in Uganda plays a crucial role in training OH professionals by incorporating OH into curricula, establishing Student OH Innovation Clubs, providing field placements, graduate fellowships, research grants, and collaborative training.
24	Tangwangvivat et al., 2019 [49]	35.Thailand, Asia	National	35. Thailand's interagency collaboration on human and animal health began with rabies prevention, expanding significantly after the 2004 avian influenza outbreak to include multiple agencies and develop a strategy for controlling zoonotic diseases, with integrated programs and capacity-building at all levels.
25	Nantima et al., 2019 [50]	32. Uganda, Africa	National	Same as 32. above
26	Kimani et al., 2019 [51]	36. Kenya, Africa	National	36. Kenya institutionalized OH in 2011, initially focusing on zoonotic diseases. In 2015, it expanded to include AMR, culminating in the 2017–2022 National Action Plan and the National Antimicrobial Stewardship Interagency Committee (NASIC), with ongoing efforts to strengthen coordination.
27	Hort et al., 2019 [18]	37.Thailand, Asia 38. Indonesia, Asia	National National	37. The extensive network of provincial, district, and subdistrict agencies, established over decades, provides a robust infrastructure for EID surveillance and response in Thailand. This intersectoral and multi-level collaboration is critical to the country's OH activities. 38. OH activities in Indonesia involve collaboration across government levels and ministries, focusing on human and animal health. Key initiatives include zoonosis control and training and expertise development.
28	Hermesh et al., 2019 [52]	39. Negev region, Israel	Local	39. OH activities in Israel's Negev region focus on addressing brucellosis, a zoonotic disease prevalent among the Bedouin Arab population. Effective control requires integrating social, political, and ethical considerations to build trust and cooperation within the OH framework.
29	de la Rocque et al., 2019 [24]	40. Multi-countries	Global	40. The IHR-MEF have enhanced and supported countries in strengthening core capacities, integrating them into national health systems, and fostering multisectoral partnerships, including animal health and environmental sectors, to promote a OH approach.
30	Bright-Ponte et al., 2019 [53]	41. USA	National	41. In the USA, multiple collaborative activities are underway to monitor antimicrobial use (AMU) and resistance, improve AMU practices across sectors, enhance understanding of AMR, slow its development, and preserve antimicrobial effectiveness, all within a OH framework.
31	Agbo et al., 2019 [54]	42. Guinea, Liberia, and Sierra Leone, Africa	Regional	42. All three countries have created National OH Platforms for multisectoral coordination, conduct annual performance assessments, and are developing updated National Preparedness & Response Plans for emerging disease threats.
32	Ghai S. & Hemachudha T., 2018 [55]	43. Six countries, Asia	Regional	43. The OH approach involves cross-sector cooperation among physicians, veterinarians, local authorities, communities, and the media. It focuses on regulations, vaccination, animal movement controls, public awareness, collaboration, resource management, and integrated surveillance to control and eliminate diseases like rabies.
33	Barroga et al., 2018 [56]	44. Bicol, Philippines	Local	44. The “Practical Inter-sectoral Linking” protocol in the Bicol Region connects local human health, animal health, and local government units for rabies detection, reporting, and intervention.
34	Sommanustweechai et al., 2017 [57]	45. Thailand, Asia	National	45. The OH approach was adopted through collaboration between national actors and international partners to better contain EIDs. It emphasizes multidisciplinary collaboration, particularly among professionals trained in Field Epidemiology Training Programs, including veterinarians and wildlife veterinarians.
35	Salyer et al., 2017 [17]	46. Multi-countries, Asia, Africa 47. Kenya, Africa	Regional National	46. Between 2014 and 2016, the US Centers for Disease Control and Prevention conducted seven OH Zoonotic Disease Prioritization workshops. These workshops enable countries to prioritize zoonotic diseases, strengthen OH collaborations, and develop action plans, even in areas lacking quantitative data. 47. Kenya, which did not plan postworkshop activities, had already created a OH strategic plan in 2012. This plan included capacity-building activities and ongoing prevention and control efforts for the prioritized zoonoses, with further support from the government of Kenya.
36	Powell et al., 2017 [21]	48. Cornwall, UK	Local	48. The OH approach in the UK, as part of NHS England's Sustainability and Transformation Plans, integrates human and animal health practices to enhance antibiotic stewardship and antimicrobial resistance strategies through education, interdisciplinary collaboration, and resource sharing.
37	Nyatanyi et al., 2017 [16]	49. Rwanda, Africa	National	49. Rwanda's OH strategic plan enhances health outcomes by integrating human, animal, and environmental sectors, supported by a robust network of community health workers and international partnerships, which accelerates response times and fosters systemic innovation.
38	Karp et al., 2017 [58]	50. USA	National	50. The National Antimicrobial Resistance Monitoring System (NARMS) monitors AMR in enteric bacteria across humans, retail meat, and food animals using a OH approach.
39	Public Health Agency of Canada. 2017 [59]	51. Canada, North America	National	51. The Pan-Canadian Framework for Action, released in 2017, employs a OH approach to combat AMR, focusing on surveillance, infection prevention, stewardship, and research to strengthen Canada's AMR response.
40	Madero et al., 2016 [27]	52. Spain, Europe 53. European countries	National Regional	52. Spain's National Plan to Combat Antibiotic Resistance implements a comprehensive, multisectoral approach across human and veterinary medicine, research, animal husbandry, and education. 53. The European Union's OH approach to combat AMR involves a comprehensive action plan developed by the European Commission, which outlines 12 key actions.
41	Degia et al., 2016 [23]	54.12 Caribbean countries, America	Regional	54. The “One Health, One Caribbean, One Love” project utilizes a collaborative and transdisciplinary OH approach across the Caribbean to enhance health outcomes by linking human, animal, plant, and environmental health. This approach increases health awareness, reduces vulnerability, boosts resilience to diseases, and improves efficiency, thereby lowering healthcare costs.
42	D'Angeli et al., 2016 [60]	55. Washington State, America	Local	55. In 2014, the Washington State Department of Health formed a OH Steering Committee and two workgroups focused on AMR. They conducted educational sessions to improve understanding of factors like antibiotic use, bacterial transmission, and environmental contamination that contribute to resistance.
43	Travis et al., 2014 [61]	56. Eastern Africa, Africa	Regional	56. The OH-related networks in Africa engage in interdisciplinary collaboration to enhance ecosystem and population health, focusing on research, surveillance, and training in infectious and transboundary animal diseases. These initiatives aim to build local capacity, advance scientific knowledge, and improve health outcomes through partnerships between African nations and global partners.
44	Shallcross et al., 2014 [28]	57. Multi-countries	Global	57. The World Health Assembly's resolution on AMR emphasizes a global action plan rooted in the OH approach, focusing on communication, infection prevention, and optimal antimicrobial and diagnostic use. It supports member states in developing national plans and enhancing capacity to combat antimicrobial resistance collaboratively across human and animal health sectors.
45	Rubin et al., 2014 [15]	58. Multi-countries 59. USA, America 60. USA, America 61. Kenya, Africa	Global National National National	58. The OH response to the HPAI H5N1 pandemic involved coordinated global surveillance, collaborative research funding, enhanced response capabilities, and improved communication between human and animal health sectors, demonstrating effective multisectoral international cooperation. 59. Coordinated surveillance involves agencies from various sectors collaborating to identify and address emerging pandemic threats by agreeing on and implementing a unified course of action to tackle urgent national issues. 60. The Foodborne Diseases Active Surveillance Network (FoodNet) is a collaborative, interagency project that provides active, population-based surveillance for nine bacterial and parasitic infections commonly transmitted through foods. The data generated by this system supports epidemiological studies, guiding public health officials on effective control measures for foodborne outbreaks. 61. The Kenya ZDU is a cross-sectoral group dedicated to zoonotic disease response, effectively integrating animal and human health expertise. The unit is co-led by epidemiologists, ensuring equal leadership and collaboration between the sectors.
46	Okello et al., 2014 [62]	62. Uganda, Africa 63. Nigeria, Africa 64. Tanzania, Africa	National	62. Established in 1992, Uganda's Coordinating Office for the Control of Trypanosomiasis (COCTU) demonstrates an early commitment to the OH approach by managing human and African trypanosomiasis. 63. Nigeria's response to the 2006 H5N1 outbreak sparked significant OH activities, enhancing collaboration between health and agriculture ministries, establishing technical committees, and promoting intersectoral partnerships. 64. In Tanzania, OH activities for rabies control highlight how localized research can shape broader policies.
47	Morgan D., 2014 [22]	65. UK	National	65. The HAIRS group coordinates activities to identify, assess, manage, and communicate zoonotic risks, with equal participation from human and animal health practitioners.
48	Mbabu et al., 2014 [63]	66. Kenya, Africa	National	66. ZDU in Kenya established in 2011, embodies a OH approach by integrating human, animal, and environmental health to manage zoonotic diseases. It includes senior epidemiologists from both health sectors and now also an ecologist to address environmental risks, enhancing disease prevention and control.
49	Jeggo M. & Mackenzie JS., 2014 [19]	67. Australia	National	67. The OH approach in Australia, demonstrated by the management of the Hendra virus, showcases the benefits of interdisciplinary collaboration in combating infectious diseases.
50	Ford J. & Nunn M., 2012 [20]	68. Brisbane, Australia	Local	68. In Brisbane, managing Hendra virus involves interdisciplinary research on the virus, bats, and horses, developing vaccines and treatments, educating on biosecurity, and coordinating efforts through an intergovernmental task force.
51	Parodi et al., 2011 [64]	69. Kazakhstan, Asia 70. Kyrgyzstan, Asia 71. Tajikistan, Asia 72. Uzbekistan, Asia	National	69. Situational analysis and capacity building to detect, diagnose, and prevent zoonotic diseases highlight opportunities to leverage regional collaboration (common socio-economic history, training and language). Intersectoral collaboration among public health services, veterinary services, and civil society groups is established at distinct and community levels. 70. Same as 69. above. 71. Same as 69. above. 72. Same as 69. above.
52	Zinsstag et al., 2009 [65]	73. Kyrgyzstan, Asia	National	73. The toolbox for the OH concept includes integrated disease surveillance, joint animal-human epidemiological studies, and collaborative health services development, aiming to foster practical cooperation and enhance global public health through interdisciplinary collaboration.
53	Mazet et al., 2009 [66]	74. Tanzania, Africa	Local	74. The HALI Project started in 2006 in rural Tanzania employs the OH approach to address zoonotic diseases and resource limitations affecting health and livelihoods in the Ruaha ecosystem. It involves testing for pathogens, monitoring water quality, assessing health impacts on pastoralist communities, introducing new diagnostics, and training locals.
54	Klement et al., 2009 [67]	75. Israel, Asia 76. Israel, Asia 77. Israel, Jordon and Palestine, Middle East	National National Regional	75. Humanized animal models for the study of infectious zoonotic diseases. 76. The OH concept enabled collaboration among veterinarians, physicians, epidemiologists, and microbiologists to better understand and prevent relapsing fever in humans and animals. 77. Avian influenza outbreaks in Israel, Jordan and the Palestinian authority – an example of national and cross boarder ‘OH’ collaboration.

**Fig. 3:**
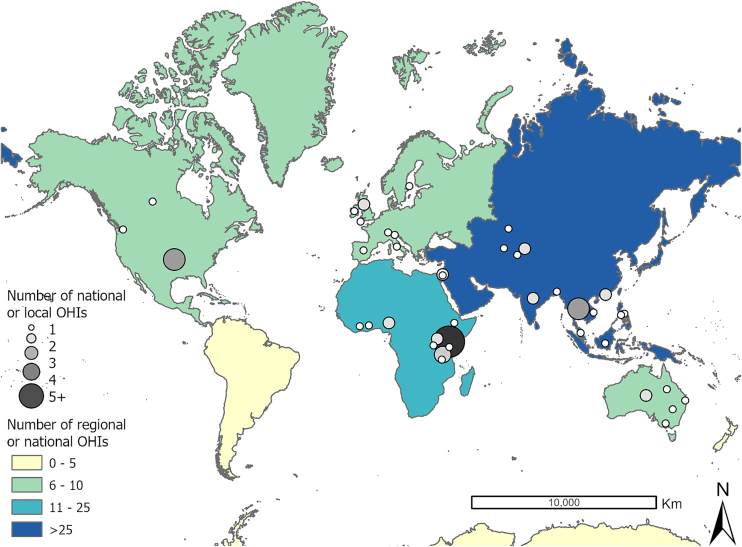
Geographical distribution of One Health implementations (OHIs) at national, local and regional levels. Note: Grey scale circles represent OHIs implemented at the national and local level, with larger and darker grey circles indicating a higher number of OH implementations. Color gradient (yellow to blue) indicates the OHIs at the regional level by world continents. International OHIs = 5.

Most OH programs operated at the national (64 %), local (17 %), and regional levels (13 %). Regional levels represent programs implemented across multiple countries within specific regions such as the Eastern Mediterranean region [26], European Union [27], and Caribbean nations [23]. These programs spanned 33 countries across five continents (Fig. 3), with most (72.7 %) implemented in the Asian and African continents. Five global programs on OH actions were identified, including programs implemented in response to emerging health threats such as HPAI [15] and antimicrobial resistance (AMR) [28]. Additionally, OH implementation aimed at strengthening multisectoral collaboration were identified across global activities, adhering to frameworks initially proposed by the Tripartite, such as the International Health Regulations (IHR) Monitoring and Evaluation Framework designed to enhance the performance of iterative evaluations and improve preparedness and response strategies for zoonotic diseases [24,29].

### 3.2 One Health activities

Table 2 shows the characteristics of OH activities across the three pathways. Among the 77 unique OH programs, 54.5 % involved activities with human and animal sectors, followed by 45.5 % across all three sectors. We found no studies involving the environment sector and its impact on human (human-environment) or animal health (animal-environment). Our analysis of stakeholder involvement in OH operational implementations has highlighted that the activities are primarily driven by government departments (94.8 %) and academic institutions (70.1 %). In contrast, there is a substantial under-representation of local communities and indigenous groups (7.8 %). Further analysis to evaluate the transparency of projects showed that the majority of OH implementations were guided by clearly defined visions and objectives (84.4 %) or expected project outputs (67.5 %). However, only half of the activities reported their named founders (49.4 %), and monitoring and evaluation strategies were described in only 44.5 % of OH programs.

**Table 2:** One Health characteristics and implementations identified in 77 unique activities mapped across three OH JPA ToC pathways.

	Frequency (n = 77)	Percentage (%)
**Sector**		
Human-animal	42	54.5 %
Human-environment	0	0
Animal-environment	0	0
All three sectors	35	45.5 %
**Stakeholders**		
Government and policy	**74**	**96.1 %**
Academic and research	**54**	**70.1 %**
Non-government organizations	33	42.9 %
Industry and private sectors	38	49.4 %
Local communities and indigenous groups	8	10.4 %
**Transparency**		
Clearly defined vision, aims or objectives	**65**	**84.4 %**
Reported named funders	38	49.4 %
Clearly stated goals or projected outputs	**52**	**67.5 %**
Monitoring and evaluation strategy	31	40.3 %
**Pathway 1: Policy, legislation, advocacy and financing**	**70**	**90.9 %**
Governance	**60**	**77.9 %**
Policy and legislation	**51**	**66.2 %**
Financing	37	48.1 %
Advocacy	40	51.9 %
**Pathway 2: Organizational development, implementation and sectoral integration**	**74**	**96.1 %**
Capacity development	**58**	**75.3 %**
Community engagement	39	50.6 %
Multisectoral coordination	**56**	**72.7 %**
Collaboration	**66**	**85.7 %**
Communication	**51**	**66.2 %**
**Pathway 3: Data, evidence and knowledge**	**70**	**90.9 %**
Scientific evidence-base	**49**	**63.6 %**
Technical tools/frameworks	43	55.8 %
Protocols and guidelines	40	51.9 %
Monitoring and evaluation strategy	42	54.5 %
Information and surveillance systems	**52**	**67.5 %**
Implementation of community-centric and risk-based solutions (e.g. vaccines)	36	46.8 %

Note: Bolded statistics indicate domains with percentages greater than 60 %.

We categorized OH activities aligned to the three interdependent pathways, each highlighting a different aspect of how OH can be achieved for planning and implementation [68].

**Pathway 1** emphasizes the foundational role of policy, legislation, advocacy, and financing that allows an enabling environment for the execution of OH activities; 90.9 % of the included programs incorporated at least one of these aspects. The majority of identified OH implementations had ‘Governance’ (77.9 %) and ‘Policy and legislation’ (66.2 %) in place, serving as the backbone for multisectoral collaboration and coordination described in pathway 2. However, only 48.1 % of activities indicated the sources of financing and investment opportunities.

**Pathway 2** underscores the operational dimensions of a OH approach. We found that the greatest proportion of activities (96.1 %) incorporated at least one dimension of capacity development, community engagement, multisectoral coordination, collaboration, or communication. Within this pathway, most dimensions were included in more than 60 % of activities, with ‘Collaboration’ reported in 85.7 % of OH activities. However, ‘Community Engagement’ was identified in only 50 % of activities.

**In pathway 3** the focus shifts to data, evidence and knowledge; ‘Information and surveillance system’ and ‘Scientific evidence-based’ approaches underpin the exchange of information and data-driven decision-making. This pathway featured in more than 60 % of OH activities. However, areas such as ‘Monitoring and evaluation strategy’ for best practices, innovation in developing ‘Technical tools/frameworks’, and the application of knowledge translation into data for evidence/technical tools/protocols/guidelines, particularly ‘Implementation of community-centric and risk-based solutions’ received relatively less attention (46.8 %).

**Fig. 4:**
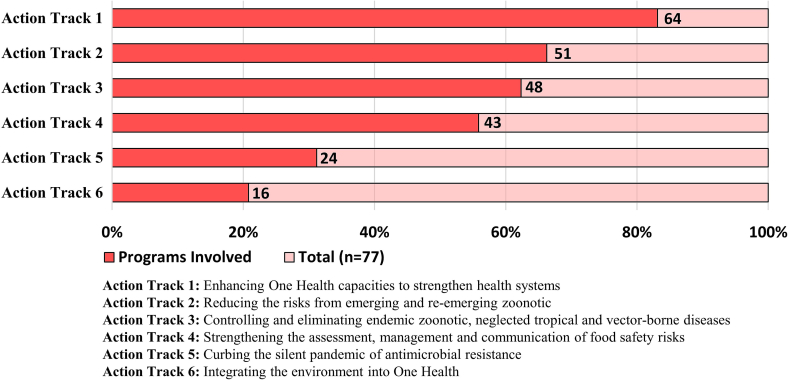
One Health implementations mapped across six action tracks.

To further discern how OH activities are associated with the specific objectives for achieving expected medium-term outcomes by 2030 outlined in the OH JPA, the included implementations (n = 77) were mapped across the six action tracks. Highlighted in Fig. 4, action track 1, which focuses on strengthening OH collaborative capacity for global coordination and high-level engagement, had the highest percentage of involvement (*n* = 64, 83.1 %) across activities. In contrast, action track 6 showed the least involvement of activities (*n* = 16, 20.8 %) integrating the environment in OH implementations. Similarly, only 24 (31.2 %) OH implementations emphasized limiting the emergence and spread of resistant pathogens through AMR control and to preserve treatment effectiveness (Action track 5). For the remaining objectives, which include reducing risks from emerging and re-emerging zoonotic pathogens (Action track 2), controlling endemic zoonotic, neglected tropical and vector-borne diseases (Action track 3), and systematically strengthening food safety efforts (Action track 4), we found that around half of OH implementations addressed these concerns.

Table 3 provides an overview of OH implementations, showing the high-level actions across the pathways contributing to the desired impact of the six action tracks (Fig. 4). High-level initiatives in action track 1 (AT1.1, AT1.2, AT1.3) were implemented in over 75 % of OH programs. These high-level actions contribute to organizational development and collaboration (Pathway 2), and knowledge creation and scientific evidence-based information systems (Pathway 3). The high-level initiatives in action track 2 (Pathway 2 and 3) aim to reduce risks from emerging and re-emerging zoonotic diseases, with a stronger emphasis on investing in surveillance systems for early detection and response (AT2.3). However, just under half of the OH programs in our review included activities focused on better understanding the drivers and spread of zoonotic pathogens (AT2.1) and incorporating knowledge to prioritize targeted interventions (AT2.2). Action track 3 aims to build awareness and demand for services to reduce the burden of endemic zoonotic, neglected tropical, and vector-borne diseases. OH implementations placed more emphasis on high-level actions (Pathway 1), focusing on strengthening policy frameworks (AT3.2) and increasing investment and political commitment (AT3.3). However, the implementation of community-centric and risk-based solutions (Pathway 3, AT3.1) to these diseases was low.

**Table 3:** One Health implementations mapped across three pathways and six action tracks (OH JPA high-level actions).

	Frequency (*n* = 77)
**Pathway 1: High-Level Actions - Policy, legislation, advocacy and financing**	
AT3.2 Strengthen policy frameworks for the control and prevention of neglected zoonotic disease	57 (74.0 %)
AT3.3 Increase political commitment and investment for control of neglected zoonotic diseases	49 (63.6 %)
*AT5.2 Reinforce global and regional initiatives to influence and support OH responses to AMR*	28 (36.4 %)
**Pathway 2: High-Level Actions - Organizational development, implementation and sectoral integration**	
*AT1.1 Establish the foundations for OH capacities*	59 (76.6 %)
AT1.2 Generate mechanisms, tools, and capacities to establish a OH competent workforce and to facilitate One Health work	58 (75.3 %)
AT1.3 Generate an enabling environment for effective implementation of OH	67 (87.0 %)
*AT2.3 Strengthen OH surveillance, early warning and response*	58 (75.3 %)
*AT4.1 Strengthen OH approach in national food controls systems and food safety coordination*	18 (23.4 %)
*AT4.2 Improve food system data and analysis, scientific evidence, and risk assessment*	17 (22.1 %)
*AT4.3 Foster the adoption of OH approach in foodborne disease surveillance systems and research*	17 (22.1 %)
*AT5.2 Reinforce global and regional initiatives to influence and support OH responses to AMR*	26 (33.8 %)
AT5.3 Strengthen global governance structures for AMR	21 (27.3 %)
AT6.1 Protect, restore and prevent ecosystem and environmental degradation	5 (6.5 %)
*AT6.3 Integrate environmental knowledge, data and evidence in decision-making*	29 (37.7 %)
AT6.4 Create an interoperable OH in-service training program for environment, medical and veterinary sector professionals	36 (46.8 %)
**Pathway 3: High-Level Actions - Data, evidence and knowledge**	
*AT1.1 Establish the foundations for OH capacities*	68 (88.3 %)
AT2.1 Understand drivers of emergence, spillover and spread of zoonotic pathogens	38 (49.4 %)
AT2.2 Identify and prioritize evidence-based upstream interventions for prevention of zoonoses	38 (49.4 %)
*AT2.3 Strengthen OH surveillance, early warning and response*	54 (70.1 %)
AT3.1 Enable countries to implement community-centric and risk-based solutions to neglected zoonotic diseases	37 (48.1 %)
*AT4.1 Strengthen OH approach in national food controls systems and food safety coordination*	21 (27.3 %)
*AT4.2 Improve food system data and analysis, scientific evidence, and risk assessment*	18 (23.4 %)
*AT4.3 Foster the adoption of One Health approach in foodborne disease surveillance systems and research*	20 (26.0 %)
AT5.1 Strengthen country capacity and capability to control AMR	25 (32.5 %)
AT6.2 Mainstream the health of the environment and ecosystems into the OH approach	19 (24.7 %)
*AT6.3 Integrate environmental knowledge, data and evidence in decision-marking*	27 (35.1 %)

Action tracks that contribute to more than one of the three pathways are illustrated by the shaded italic.

The high-level actions related to action tracks 4 and 5 across all pathways which focus on assessment and management of food safety risks and limiting emergence and spread of AMR, respectively, showed moderate implementation of between 20 and 40 %. The high-level actions that aim to integrate the environment into OH (Pathway 1, 2 and 3, Action track 6) were the least mentioned. Notably, actions designed to protect the environment and prevent its further degradation (AT6.1) had the lowest implementation at 6.5 %.

## 4 Discussion

Our scoping review identified and described OH strategies and programs, enabling evaluation of the implementation of OH from local communities to global initiatives. Using a novel approach, we applied the OH JPA ToC which provided a framework to classify, categorize and map OH implementation against the established three pathways for change and the six action tracks. This allowed us to identify existing gaps in OH initiatives described in peer reviewed literature, providing indications of actions required to strengthen and operationalize OH. Differences in OH implementation may be indicators of insufficient capacity and capability in some areas, indicating the need for holistic strengthening of health systems to ensure the sustainable changes required for achieving the medium and long-term outcomes outlined in the OH JPA. Conversely, the differences may concern limitations with implementing the OH JPA ToC framework in failing to recognize or consider institutional factors for operationalizing OH.

A key finding from our scoping review indicates that OH implementations have been heavily focused on collaboration between human and animal sectors, with minimal engagement of the environment sector. Only one in five OH initiatives integrated environmental concerns, such as climate change and impact on ecosystems. This is demonstrated in examples such as the historical integration and collaboration of human-animal sectors in zoonotic disease response, despite needed engagement with climate impacts on biodiversity and ecosystems which drive disease emergence. Governance challenges of working in silos rather than across sectors, inadequate understanding of the role of the environment and its determinants in OH may hinder the inclusion of the environment in operationalizing OH [69,70]. Involvement of the environment sector could have great potential to increase the efficacy of previous OH efforts and yield substantial co-benefits [6,11]. Given the growing threats posed by climate change, habitat destruction, and biodiversity loss, it is imperative to adopt a comprehensive approach that integrates the environmental dimensions of health.

Our analysis further revealed that OH activities to support AMR control, including stewardship of antibiotic use across sectors to preserve treatment effectiveness, were reported far less often than activities in other domains. AMR is one of the leading global health challenges, is described as a OH problem requiring urgent action to tackle the emergence and spread of resistant pathogens within and between the three sectors [71]. Targeted OH interventions supported by robust intersectoral collaborations to reduce the burden of AMR will lessen its threat to global health security. Integrated environmental surveillance, national action plans that acknowledge coordinated OH activities to implement solutions and advocating for global action to mitigate impacts of AMR through a OH lens is required [72]. Incorporation of environmental considerations into AMR strategies and broader OH frameworks and initiatives ensure countries can better address emerging health challenges and promote ecosystem health and resilience [69].

We found a greater representation of pathway 1 and pathway 2 implementation highlighting the positive and necessary elements for operationalizing OH. Pathway 1 emphasizes the foundational building blocks required to support effective OH implementation, as evidenced by a higher proportion of activities that incorporated policy, legislation, advocacy, and financing. Operationalizing OH requires a health in all policy approach and good governance promoting stakeholder participation, transparent decision making and political support to create and enforce regulations and policies that support integrated OH approaches. Improving the capacity of OH governance is critical for effective and sustainable OH policy and practice [73]. Pathway 2 underscores the operational dimensions of OH, and in our review, most OH activities. These are predominantly focused on human and animal health, incorporating at least one dimension of capacity development, community engagement, multisectoral coordination, collaboration and communication. This highlights the positive steps taken towards building capacity and engaging collaboration for successful implementation of OH interventions [74,75].

Many included studies reported on OH implementation activities following major global health crises such as SARS (2003), H1N1 influenza (2009), Middle East respiratory syndrome (2012), Ebola virus disease (2014) and more recently COVID-19 [76]. This evokes a reactive focus on preparedness and crisis response rather than prevention [69]. While it is essential to have robust mechanisms in place for detecting and responding to health crises, greater emphasis should be placed on preventing the emergence and spread of infectious diseases and other health threats through managing anthropogenic environmental and human health impacts [77]. Preparedness involves investing in early warning systems, strengthening surveillance and monitoring efforts, and preventive interventions such as vaccination campaigns, vector control measures, and public health awareness programs [78,79]. Shifting the focus towards proactive and preventive measures is more cost effective. For example, it is estimated that expenses associated with preventing viral zoonoses are substantially lower than those incurred in disease management and the economic impact of loss-of-life [8,9]. By prioritizing prevention and preparedness, countries can reduce the burden of disease, minimize health risks, and enhance overall resilience to health emergencies.

Few studies reported involvement from local communities and Indigenous peoples on OH implementation. This is despite the acknowledgement of their critical role as key engagers and stakeholders across all three pathways. OH operationalization continues to be driven by higher level policy and governance as advocated in pathway 1. While government departments and academic institutions play significant roles in planning OH activities, they are often focused on the ‘what and why’ of OH implementation, rather than the ‘how’. Community stakeholders often possess valuable insights, knowledge, and resources that are essential for the successful contextualization, implementation and sustainability of OH initiatives, particularly at the grassroots level [80]. A systematic review of the barriers and enablers to OH activities in LMIC identified the need for more engagement of local communities to ensure their involvement in the implementation of OH programs [81,82]. Therefore, efforts should be made to actively engage, empower and collaborate with local communities, Indigenous peoples and other stakeholders in the planning, implementation, and evaluation of OH programs. Inuit communities have demonstrated success with OH operationalization supported by inclusion of social science research, decision-making with and by local communities, and building on existing long-lasting relationships with organizations [83]. Appropriate and relevant OH models for local and indigenous communities should explicitly engage all relevant stakeholders through a bottom-up and top-down approach, with activities including fostering partnerships, promoting participatory approaches, and ensuring representation and inclusivity in decision-making processes [84].

Over half of OH projects were funded or led by researchers from high-income countries. Many of these projects were carried out in LMIC and primarily focused on infectious diseases. There is considerable variation in OH implementation between countries, with LMIC supported by wider international programs. Greater focus on OH in LMIC can suggest differing health priorities with greater needs because of infectious diseases, environmental impacts, health inequities, political will and low resource availability. On the other hand, high-income countries are concerned with pandemic risks, potentially overlooking the need to implement OH projects in their own jurisdictions to address issues such as environmental destruction, climate change and pollution which affect their own health [73]. Few studies report long-term evaluations of sustainability and cost effectiveness of interventions. Embedding capacity development into OH projects ensures sustainability as identified in a systematic review of barriers and enablers to OH activities in developing countries [82].

Less than half of the OH programs in our review indicated sources of financing and investment opportunities. Adequate financial resources are needed to support activities, build capacity, and maintain essential infrastructure and services for ensuring sustainable implementation of OH. Investments should be prioritized based on the potential impact, cost-effectiveness, and sustainability of OH interventions, with a focus on addressing the most pressing health challenges and promoting equity and social justice. Additionally, a limited number of studies provided cost-effectiveness data or monitoring and evaluation strategies of OH programs. These should be embedded into project planning and implementation so that the benefit of OH initiatives can be clearly recognized. Without long-term evidence of added value, policy makers are unlikely to invest resources in OH activities [85]. Furthermore, examples showing evidence on practical implementation of OH principles at the interface of human, animal, and environmental health remains limited, highlighting the need for detailed case studies that delineate specific actions taken and suggested improvements for this ongoing process.

### 4.1 Limitations

Our study was based on published peer-reviewed literature in English, which may potentially overlook valuable insights from non-English speaking regions and broader grey literature outside of the Quadripartite collaboration reports. Publication bias may lead to an incomplete understanding of the global landscape of OH implementation. Acknowledging this, future studies could benefit from incorporating a more diverse array of sources, including primary data collection from grey literature and non-English case studies. Enabling a deeper insight of nuanced regional and country-specific challenges will offer a comprehensive view of global OH implementation. Additionally, our analysis does not provide an in-depth examination of individual country strategies and policies and reports outside of the OH JPA ToC framework, nor does it include an economic evaluation of OH implementations due to data availability. Further research is warranted on detailed reviews of national strategies to uncover challenges and successes in OH implementations at national and local levels. It is also important to provide policymakers with crucial data on resource allocation and cost-effectiveness of OH implementations, facilitating informed decisions that consider regional, country-specific, and local characteristics to justify OH investments.

### 4.2 Future directions for OH operationalization

Future directions for OH operationalization should refer to a stepwise approach, emphasizing a cycle of continuous improvement in implementation [68]. For countries and regions that have adopted the OH approach, ongoing monitoring and evaluation are crucial for tracking progress and making necessary adjustments [24,29]. A fundamental step in planning and enhancing OH implementation is conducting a situational analysis to understand the current state of OH efforts [86,87]. This involves mapping relevant stakeholders and/or reviewing OH-related results. Strengthening multisectoral OH coordination mechanisms is essential to ensure effective collaboration among diverse sectors [6,22]. Subsequently, developing a national OH action plan with prioritized activities and clearly defined responsibilities for stakeholder groups is needed [21]. Robust execution of the action plan, supported by continuous feedback loops, would not only allow for adjusting activities over time but also align better with the dynamic nature of challenges to enhance both the adaptiveness and effectiveness of the OH approach. Finally, a comprehensive review and sharing of lessons learned will foster continuous improvement [68]. By following these structured steps, countries can effectively operationalize OH principles, ensuring a coordinated and holistic approach to foster sustainable health systems and improve the well-being of all populations.

## 5 Conclusion

This study enhances empirical knowledge by systematically analysing how previous OH activities have been implemented. The findings identify gaps in current practices that are necessary for achieving sustainable change and optimizing future OH operations reflecting uneven implementation of OH actions across the different OH JPA ToC pathways and action tracks. The action tracks emphasize that there is a systems approach to reducing health threats and that their interdependence across the human, animal and environment is clear. While there is commendable progress in enhancing OH capacities and strengthening surveillance systems, there are notable gaps in understanding zoonotic disease drivers, community-centric solutions, food safety, AMR, and environmental integration. Inequity in the system results in an imbalance which consequently impacts outcomes as reflected in the findings from our review identifying a low proportion of programs, particularly within action tracks 5 (global and regional governance) and 6 (health of the environment) which has implications for achieving the desired outcomes. By incorporating best practices and refining strategies for growing OH activities, we can maximize capabilities for OH implementation at every level and be better equipped to prevent, predict, detect, and respond to global health threats.

## Funding

The project was funded by The University of Adelaide Faculty of Health and Medical Sciences, Strategic Research Support Scheme and the Environment Institute.

## CRediT authorship contribution statement

**Adriana Milazzo:** Writing – review & editing, Writing – original draft, Supervision, Resources, Project administration, Methodology, Investigation, Conceptualization. **Jingwen Liu:** Writing – review & editing, Writing – original draft, Visualization, Methodology, Investigation, Formal analysis, Data curation. **Priyanka Multani:** Writing – review & editing, Methodology, Investigation, Formal analysis, Data curation, Conceptualization. **Sandra Steele:** Writing – review & editing, Methodology, Investigation, Data curation, Conceptualization. **Elizabeth Hoon:** Writing – review & editing, Conceptualization. **Anne-Lise Chaber:** Writing – review & editing, Writing – original draft, Supervision, Methodology, Investigation, Funding acquisition, Data curation, Conceptualization.

## Declaration of competing interest

The authors declare no competing interests.

## Data Availability

Data will be made available on request.

## Appendix A Supplementary data

Supplementary material Image 1
