# Supplementary material for: One Health implementation: A systematic scoping review using the Quadripartite One Health Joint Plan of Action
Source: One Health. 2025 Mar 2;20:101008. doi: 10.1016/j.onehlt.2025.101008 (PMC11953970; doi:10.1016/j.onehlt.2025.101008)
Supplement: Supplementary file 1 — Supplementary material [file mmc1.docx]

**Appendix A**

**Table A1. Search strategy: terms, databases, limitations and number of articles for review.**

| **Databases** | **Strategy** | **Number of hits** | **Number imported into Endnote** |
| --- | --- | --- | --- |
| PubMed  Filters:  English, Full text  Date (publication):  ~June 2023 | #1 Operationalis*[tiab] OR Operationaliz*[tiab] OR Implement*[tiab] OR Action*[tiab] OR enforcement[tiab] OR application*[tiab] OR practice*[tiab] OR operation[tiab] OR Strateg*[tiab] OR Approach*[tiab] OR Intervention*[tiab] OR Barrier*[tiab] OR Facilitat*[tiab] OR Challenge*[tiab] OR Enabler*[tiab] OR Impediment*[tiab] OR multi-sectoral[tiab] OR communication*[tiab] OR collaborati*[tiab] OR coordination*[tiab] OR capacity building[tiab] OR capacity strengthening[tiab] OR economic benefit*[tiab] OR "Health Plan Implementation"[mh]  #2 "Planetary health"[tiab] OR "Ecosystem health"[tiab] OR "One medicine"[tiab] OR "One Health"[tiab] OR "One Health"[mh]  #3 Veterinary[tiab] OR "animal health"[tiab] OR "human health"[tiab] OR environment*[tiab] OR "Environment"[mh] OR "Veterinary Medicine"[mh]  #4 Legislati*[tiab] OR Law[tiab] OR Laws[tiab] Legal[tiab] OR Act[tiab] OR Acts[tiab] OR Rule*[tiab] OR Regulat*[tiab] OR govern*[tiab] OR polic*[tiab] OR "Policy"[mh] OR "Policy Making"[mh] OR "Legislation as Topic"[mh]  #1 AND #2 AND #3 AND #4 | 8.073,237  11,901  2,369,752  3,270,066 | 1,153 |
| Embase  Filters:  English, article  Date (publication):  ~June 2023 | #1 Operationalis*.ti,ab OR Operationaliz*.ti,ab OR Implement*.ti,ab OR Action*.ti,ab OR enforcement.ti,ab OR application*.ti,ab OR Approach*.ti,ab OR practice*.ti,ab OR operation.ti,ab OR Strateg*.ti,ab OR Intervention*.ti,ab OR Barrier*.ti,ab OR Facilitat*.ti,ab OR Challenge*.ti,ab OR Enabler*.ti,ab OR Impediment*.ti,ab OR multi-sectoral communication.ti,ab OR collaborati*.ti,ab OR coordination*.ti,ab OR capacity building.ti,ab OR capacity strengthening.ti,ab OR economic benefit*.ti,ab  #2 Planetary health.ti,ab OR Ecosystem health.ti,ab OR One medicine.ti,ab OR One Health.ti,ab OR Exp *One Health/  #3 Veterinary.ti,ab OR animal health.ti,ab OR human health.ti,ab OR environment*.ti,ab OR Exp *Environment/ OR Exp *Animal Health/  #4 Legislati*.ti,ab OR Law.ti,ab OR Laws.ti,ab OR Legal.ti,ab OR Act.ti,ab OR Acts.ti,ab OR Rule*.ti,ab OR Regulat*.ti,ab OR govern*.ti,ab OR polic*.ti,ab OR Exp *Public Policy/ OR Exp *Law/  #1 AND #2 AND #3 AND #4 | 3,034,485  3,773  862,541  864,518 | 1,185 |
| Scopus  Filters:  English, Full text  Date (publication):  ~June 2023 | #1 operationalis* OR operationaliz* OR implement* OR action* OR enforcement OR application* OR practice* OR operation OR strateg* OR approach* OR intervention* OR barrier* OR facilitat* OR challenge* OR enabler* OR impediment* OR "multi-sectoral OR Communication" OR collaborati* OR coordination* OR "capacity building" OR "capacity strengthening" OR "economic benefit*"  #2 "Planetary health" OR "Ecosystem health" OR "One medicine" OR "One Health"  #3 Veterinary OR “animal health” OR “human health” OR environment*  #4 legislati* OR law OR laws OR legal OR act OR acts OR rule* OR regulat* OR govern* OR polic*  #1 AND #2 AND #3 AND #4 | 57,449,217  144,523  20,562,752  25,404,000 | 2,590 |
| **Total results** |  |  | **4,928** |

**Table A2. Detailed reasons for exclusion of studies (n=49) after full-text screening**

| No. | Study | Reasons |
| --- | --- | --- |
| 1 | (Meisser, Schelling, and Zinsstag 2011) | This study investigates the opportunities for implementation of the OH concept in Switzerland. |
| 2 | (Leung, Middleton, and Morrison 2012) | This study discusses how public health actors in Ontario are influenced by the holistic principles of OH and EcoHealth. |
| 3 | (Plumb, Olsen, and Buttke 2013) | Advocate for a OH approach to brucellosis management instead of focusing on practical implementation. |
| 4 | (Cleavel, Borner, and Gislason 2014) | This paper highlights the contributions of ecology and conservation to OH and identifies four key areas for practical engagement, emphasizing ecological and conservation goals. |
| 5 | (Flory 2014) | This study lacks a direct connection to practical experience. |
| 6 | (Nabarro and Wannous 2014) | The authors advocate for incorporating the OH approach into national health policies, education for medical and veterinary students, and disaster preparedness planning. |
| 7 | (Romanelli, Cooper, and de Souza Dias 2014) | The authors discuss, rather than implement, OH, focusing on the relationship between biodiversity, health, and policy development. |
| 8 | (Bartholomew et al. 2015) | The emphasis is on strategies for decision-makers and scientists to provide a sustainable biological science program supporting the OH initiative. |
| 9 | (Hyatt et al. 2015) | Suggestions rather than implementations. |
| 10 | (Gostin and Katz 2016) | This study proposed a series of recommendations focus on the development and strengthening of International Health Regulations (IHR) core capacities. |
| 11 | (Jacobsen et al. 2016) | Perspectives: Lessons learned from the Ebola outbreak. |
| 12 | (Queenan, Häsler, and Rushton 2016) | Proposes a conceptual framework for a OH approach to AMR surveillance. |
| 13 | (Willingham et al. 2016) | Participants in workshops suggested a framework for practicing OH in the Caribbean. |
| 14 | (Ladeira, Frazzoli, and Orisakwe 2017) | One Health is suggested to address the burden of non-communicable diseases. |
| 15 | (Queenan et al. 2017) | The paper highlights sectionalized health systems, the need for integration, and the Sustainable Development Agenda 2030 as an opportunity for change. |
| 16 | (Rüegg et al. 2017) | The paper outlines a conceptual framework for OH from a workshop by the “Network for Evaluation of One Health” but lacks actual OH implementations. |
| 17 | (Balkhy et al. 2018) | Roundtable discussion paper, addresses AMR and promotes PH concept. |
| 18 | (Degeling et al. 2018) | Adds nuance to OH research on rabies risks in northern Australia but lacks implementation details. |
| 19 | (Hitziger et al. 2018) | Proposes knowledge integration for OH governance and suggests promotion methods but lacks implementation details. |
| 20 | (Berger et al. 2019) | Identifies gaps in emergency information-sharing and explores improvements but lacks OH implementation. |
| 21 | (Hern et al. 2019) | This review updates information on AMR and proposes strategies but lacks OH implementation. |
| 22 | (Kumar et al. 2019) | Panel’s discussion rather than OH implementation. |
| 23 | (Pelican et al. 2019) | This paper presents a framework on aligning commonly implemented OH tools to support countries. |
| 24 | (Thakur and Gray 2019) | Editorial perspective rather than OH implementation. |
| 25 | (Aggarwal and Ramachandran 2020) | Describes OH criteria and a protocol for vector-borne diseases but lacks implementation details. |
| 26 | (Alam et al. 2020) | The Pearl River Declaration calls for establishing a OH Cooperation Network in Southeast Asia–Pacific, aiming to strengthen regional health security. |
| 27 | (Isk et al. 2020) | Examines drivers of AMR and proposes OH strategies but lacks OH implementation. |
| 28 | (Lindahl et al. 2020) | Workshop results suggest collaborative OH strategies for controlling brucellosis in veterinary and human medicine, rather than detailing implementation. |
| 29 | (Mardones et al. 2020) | Perspective research on sustainable OH pathways rather than implementation. |
| 30 | (Abuzerr, Zinszer, and Assan 2021) | Suggestions to improve an integrated OH surveillance system to control zoonotic diseases, but no implementation. |
| 31 | (Aguirre et al. 2021) | This study highlights cases illustrating gaps in understanding the illegal wildlife trade and zoonotic disease transmission, arguing for more integrative science using the OH approach. |
| 32 | (de la Rocque et al. 2021) | Highlights the need for concrete foundations of multisectoral coordination in international frameworks for evaluation, but no implementation. |
| 33 | (Hulme 2021) | Presents a preliminary roadmap to One Biosecurity, outlining advantages and challenges to stimulate debate, but lacks implementation details. |
| 34 | (Bronzwaer et al. 2022) | Conference session documenting challenges to interagency cooperation and solutions, but no implementation details. |
| 35 | (Dente et al. 2022) | This paper focuses on the development of a conceptual framework using existing prevention and preparedness plans. |
| 36 | (Fasina et al. 2022) | Discussion on prioritization for applied and implementation OH research. |
| 37 | (He et al. 2022) | This paper offers a scoping analysis and discussion for the OH approach within zoonosis, without providing clear OH implementation case studies. |
| 38 | (Ho 2022) | This paper offers a recommendation rather than being implemented. |
| 39 | (Ogunseitan 2022) | Opinion paper—advisory, not OH implementation |
| 40 | (Song et al. 2022) | Promoting OH concept |
| 41 | (A. et al. 2023) | Case studies provide recommendations rather than demonstrating OH implementation. |
| 42 | (Hailat et al. 2023) | Roundtable discusses priority areas but does not address OH implementation |
| 43 | (Humboldt-Dachroeden 2023) | Survey discussion, not OH implementation. |
| 44 | (T. et al. 2023) | This paper is excluded because it discusses weaknesses in frameworks and is not implemented. |
| 45 | (Allen 2015) | Discuss only the intersection of governance and OH, and do not include any information about implementation. |
| 46 | (Connolly 2017) | This article presents a framework for integrating knowledge across public and animal health sectors, focusing on discussion rather than program implementation. |
| 47 | (Wilcox et al. 2019) | Excluded for lack of information on previous implementation: describes operational criteria for OH with a protocol currently tested for vector-borne disease interventions. |
| 48 | (Asaaga et al. 2021) | Focuses on discussing policy coherence rather than specific OH implementation. |
| 49 | (Ghai and Hemachudha 2018) | Examples are provided to demonstrate OH advocacy rather than actual implementation. |

**Figure A1. Data Extraction Template**
